# Acute Pesticide-Related Illness Resulting from Occupational Exposure to Acrolein — Washington and California, 1993–2009

**Published:** 2013-04-26

**Authors:** Luis Rodriguez, Joanne Prado, April Holland, John Beckman, Geoffrey M. Calvert

**Affiliations:** Washington State Dept of Health; California Dept of Pesticide Regulation; California Dept of Public Health; Div of Surveillance, Hazard Evaluations, and Field Studies, National Institute for Occupational Safety and Health, CDC

Acrolein is an aquatic herbicide used in the western United States to prevent impaired water flow in irrigation canals. Despite its toxicity, few cases of acrolein-related illness have been reported in the literature. On August 15, 2012, an irrigation district notified the Washington State Department of Labor & Industries (L&I) of acrolein-related illness in one of its pesticide applicators. L&I inspected the site and interviewed the exposed worker, coworkers, and employer. The Washington State Department of Health assisted by obtaining medical records, interviewing the patient and hospital staff, and reviewing information obtained from L&I. To look for additional cases, CDC reviewed data from the SENSOR-Pesticides program* and the California Department of Pesticide Regulation for 1993–2009, the most recent years of data availability, and identified seven additional cases of acute acrolein-related illness.

A licensed aquatic pesticide applicator aged 57 years, previously healthy and employed for 15 years by an irrigation district in Washington, was exposed to acrolein while monitoring an application† to an irrigation canal in the Quincy-Columbia Basin. The man was not wearing the label-required respiratory protection, gloves, or a long-sleeved shirt§ when he investigated a leak in the connection between the acrolein tank and the metal assembly through which acrolein flows.

Almost immediately after exposure to the leak the worker had burning, watery eyes. Within 2 hours he experienced throat tightness, difficulty breathing, inability to swallow, moderate phlegm production, vomiting, and inability to talk because of dyspnea. He was admitted to the intensive-care unit and approximately 6 hours after exposure developed right facial droop but no other weakness or paresthesias. Supportive treatment was provided, including administration of epinephrine. Approximately 48 hours after exposure, the patient went into ventricular fibrillation and concomitantly experienced a grand mal seizure. His condition was ultimately stabilized, and he was discharged to home after a 3-week hospitalization. He received a diagnosis of lateral medullary syndrome and continued to have dysphagia, right-sided facial droop, and left-sided altered thermal skin sensitivity. He returned to work at the irritation district for 1 month in January 2013 but is not currently working because of ongoing medical conditions.

CDC identified seven additional cases of acute acrolein-related illness in the United States during 1993–2009, all in California. Five cases were among workers employed by irrigation districts, of whom four were pesticide applicators and one maintained pesticide application equipment. Six of the workers were men, and the mean age was 41 years (range: 24–53 years). Four workers had low severity illness, and three had illness of moderate severity.¶ Common symptoms were eye irritation (five workers), headache (three), dyspnea (two), and skin irritation or burns (two). No worker was hospitalized, but two lost time from work.

Acrolein is highly volatile, producing an extremely irritating vapor that is highly reactive and acts by degrading cellular structures by cross-linking proteins (1). Acrolein also can produce inflammation of the heart, and ventricular fibrillation can occur in the setting of epinephrine administration combined with an acrolein-induced catecholamine release (2). Although acrolein is measureable in blood and urine, these tests are not commonly available and are not useful in assessing exposure (3).

Because of its toxicity, acrolein is applied only through closed systems, which prevents its release into the air. Such systems are not closed during set up and break down, and visual inspection of application equipment can involve exposure to leaks; therefore, applicators must comply with stringent requirements for personal protective equipment (PPE) when performing these activities (1). Use of a closed application system combined with annual training, applicator certification,** adherence to the manufacturer’s other operating procedures for acrolein (4), and compliance with PPE requirements are expected to effectively prevent exposures of concern to workers (1). Use of nonchemical means to prevent clogging of irrigation canals with weeds and algae (e.g., mechanical harvesting, sediment removal, canal lining, and replacing the canal with piping) have been considered by irrigation districts in Washington but found not feasible because of cost and the potential for increased risk for injury to workers (5).

## Footnotes

* Additional information available at http://www.cdc.gov/niosh/topics/pesticides/overview.html.

† Magnacide H Herbicide, Baker Petrolite Corporation, EPA registration number 10707-9.

§ Label-required PPE includes a long-sleeved shirt and long pants, shoes, and socks, chemical-resistant gloves made of butyl rubber, and a National Institute of Occupational Safety and Health–approved full-face respirator with either an organic-vapor-removing cartridge with a prefilter approved for pesticides or a canister approved for pesticides.

¶ Severity of illness and injury of cases can be categorized into four groups using standardized criteria for state-based surveillance programs: low, moderate, high, and death. In low severity cases, illness/injury usually resolves without treatment and <3 days are lost from work. Moderate severity cases involve non–life-threatening health effects that are generally systemic and require medical treatment. No residual disability is detected, and time lost from work is ≤5 days. Additional information available at http://www.cdc.gov/niosh/topics/pesticides/pdfs/pest-sevindexv6.pdf.

** Additional information available at http://www.epa.gov/oppfead1/safety/applicators/applicators.htm.

## References

[1] US Environmental Protection Agency (2008). Reregistration eligibility decision document for acrolein.

[2] Beauchamp RO, Andjelkovich DA, Kligerman AD, Morgan KT, Heck HD (1985). A critical review of the literature on acrolein toxicity. Crit Rev Toxicol.

[3] Agency for Toxic Substances and Disease Registry (2007). Toxicological profile for acrolein.

[4] Baker Petrolite (2005). Magnacide H herbicide application and safety manual.

[5] Washington State Department of Ecology (2012). Fact sheet for the irrigation system aquatic weed control National Pollutant Discharge Elimination System (NPDES) and state waste discharge general permit.

